# Application of adaptive deep learning-based automatic segmentation in radiomics model for preoperative WHO/ISUP grading of clear cell renal cell carcinoma: a retrospective comparative study with manual segmentation

**DOI:** 10.7717/peerj.21022

**Published:** 2026-03-27

**Authors:** Hongqing Zhu, Zhihui Chen, Jianbo Zhang, Moran Yang, Kangchen Gu, Wenxia Bao, Yinlai Du, Sihui Hou, Wenjun Yao

**Affiliations:** 1 The Second Affiliated Hospital, Anhui Medical University, Hefei, China; 2 Medical Imaging Research Center, Anhui Medical University, Hefei, China; 3 Electronic Information Engineering, Anhui University, Hefei, China; 4 The Second Clinical Medical School, Anhui Medical University, Hefei, China

**Keywords:** Automatic segmentation, Clear cell renal cell carcinoma, World Health Organization/International Society of Urological Pathology, Radiomics, Tumor grading prediction

## Abstract

**Aim:**

To evaluate the effectiveness of different methods for segmenting tumor regions of interest in building prediction models for the World Health Organization/International Society of Urological Pathology (WHO/ISUP) grade of clear cell renal cell carcinoma (ccRCC).

**Materials and Methods:**

This retrospective single-center study analyzed computed tomography (CT) images of 405 patients (training/test cohort, 324/81) with pathologically confirmed ccRCC. Two methods were used for tumor segmentation: (1) automatic segmentation: the nnU-Net model, trained on the public KiTS19 dataset, and (2) manual segmentation. Radiomics features were extracted and selected from both automatically and manually segmented images. Support vector machine (SVM) and K-nearest neighbors were used to construct pathological grade prediction models. The segmentation accuracies of nnU-Net and manual annotation were compared. The receiver operating characteristic curve, area under the curve (AUC), accuracy, sensitivity, and specificity were used to evaluate diagnostic performance. The DeLong test was used to assess the differences between the models.

**Results:**

The average Dice similarity coefficient was 0.842 ± 0.149. Automatic segmentation was time-efficient. A total of 1,834 features were extracted from each tumor. The AutoSeg-SVM model with nine features achieved the highest diagnostic performance, with an AUC value of 0.865 (0.726–1.000), an accuracy of 79.0%, sensitivity of 85.7%, and specificity of 77.6%. Both models in the automatic segmentation group showed comparable or slightly better performance than those in the manual segmentation group, although the differences were not statistically significant.

**Conclusions:**

The nnU-Net automatic segmentation provided diagnostic efficacy comparable to manual segmentation in preoperative WHO/ISUP grade prediction for ccRCC. It significantly reduced the time required for lesion segmentation and improved workflow efficiency.

## Introduction

According to the 2022 Global Cancer Statistics, there were 434,419 new cases of kidney cancer and 155,702 kidney cancer-related deaths worldwide (Bray et al., 2024). Renal cancer has diverse histological classifications, with approximately 75% of cases being clear cell renal cell carcinoma (ccRCC) (Ljungberg et al., 2022). The 2016 World Health Organization/International Society of Urological Pathology (WHO/ISUP) grade system classifies ccRCC into four grades, with grade I and II categorized as low-grade tumors and grade III and IV as high-grade tumors (Delahunt et al., 2019; Moch et al., 2016). Studies have shown that pathological nuclear grading is an independent risk factor for tumor malignancy and postoperative recurrence (Dagher et al., 2017; El Khoury et al., 2021; Kato et al., 2023). Preoperative evaluation of tumor tissue pathological grading plays a crucial role in assessing malignancy, tailoring treatment approaches, and forecasting patient prognosis (Kim et al., 2018). Percutaneous biopsy is an invasive procedure commonly used for the preoperative diagnosis of renal tumors (Blumenfeld et al., 2010; Kutikov et al., 2016). It may lead to complications, such as tumor spread, bleeding, and infection, and due to tumor heterogeneity, biopsy may underestimate tumor grading (Millet et al., 2012; Sanchez, Feldman & Hakimi, 2018).

Radiomics, by transforming imaging data into a large amount of quantifiable data, delves into underlying lesion characteristics, offering potential advancement in tumor grade prediction research (Cellina et al., 2024; Zheng et al., 2021). The traditional radiomics workflow consists of dataset construction, tumor segmentation, feature extraction, and model development (Gillies, Kinahan & Hricak, 2016). Manual tumor segmentation by radiologists is time-consuming, labor-intensive, and susceptible to subjective factors. Convolutional neural networks are efficient and objective methods for tumor segmentation (Magnuska et al., 2024). The nnU-Net framework is based on the U-Net architecture. Its main advantage is to automatically set up the segmentation process for a given dataset, which avoids extensive manual tuning. Therefore, we selected nnU-Net as our core segmentation model. Previous research on tumor segmentation primarily focused on improving segmentation accuracy (Feng et al., 2022; Houshyar et al., 2021; Hu et al., 2024). However, regardless of the segmentation method used, the final grading results are the most important for clinicians. Therefore, if an automatic segmentation method with high accuracy can be used without affecting the grading results, it will meet clinical needs, significantly reduce manual lesion delineation labor costs, and optimize workflow.

In this study, a deep learning-based automatic segmentation model was used to segment ccRCC tumors and compare the results with the radiologist’s manual delineation. The aim of this study was to assess whether automatic segmentation *vs*. manual delineation affects the WHO/ISUP grade prediction for ccRCC based on radiomics. While automatic segmentation has been explored in radiomics for various cancers, direct comparisons of automatic *vs*. manual segmentation specifically for preoperative grading of ccRCC are limited, making this study a valuable contribution.

## Materials and Methods

### Patients

**Figure 1:**
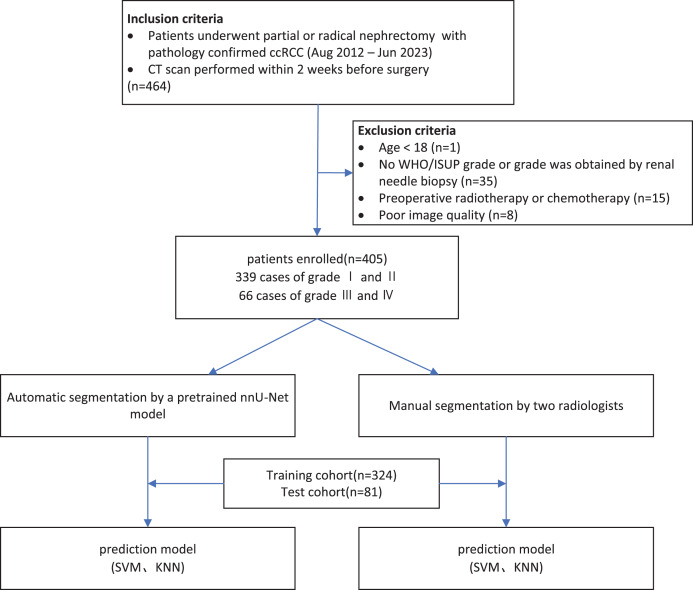
The patient recruitment pathway. WHO/ISUP, World Health Organization/International Society of Urological Pathology; SVM, Support Vector Machine; KNN, K-Nearest Neighbors.

**Figure 2:**
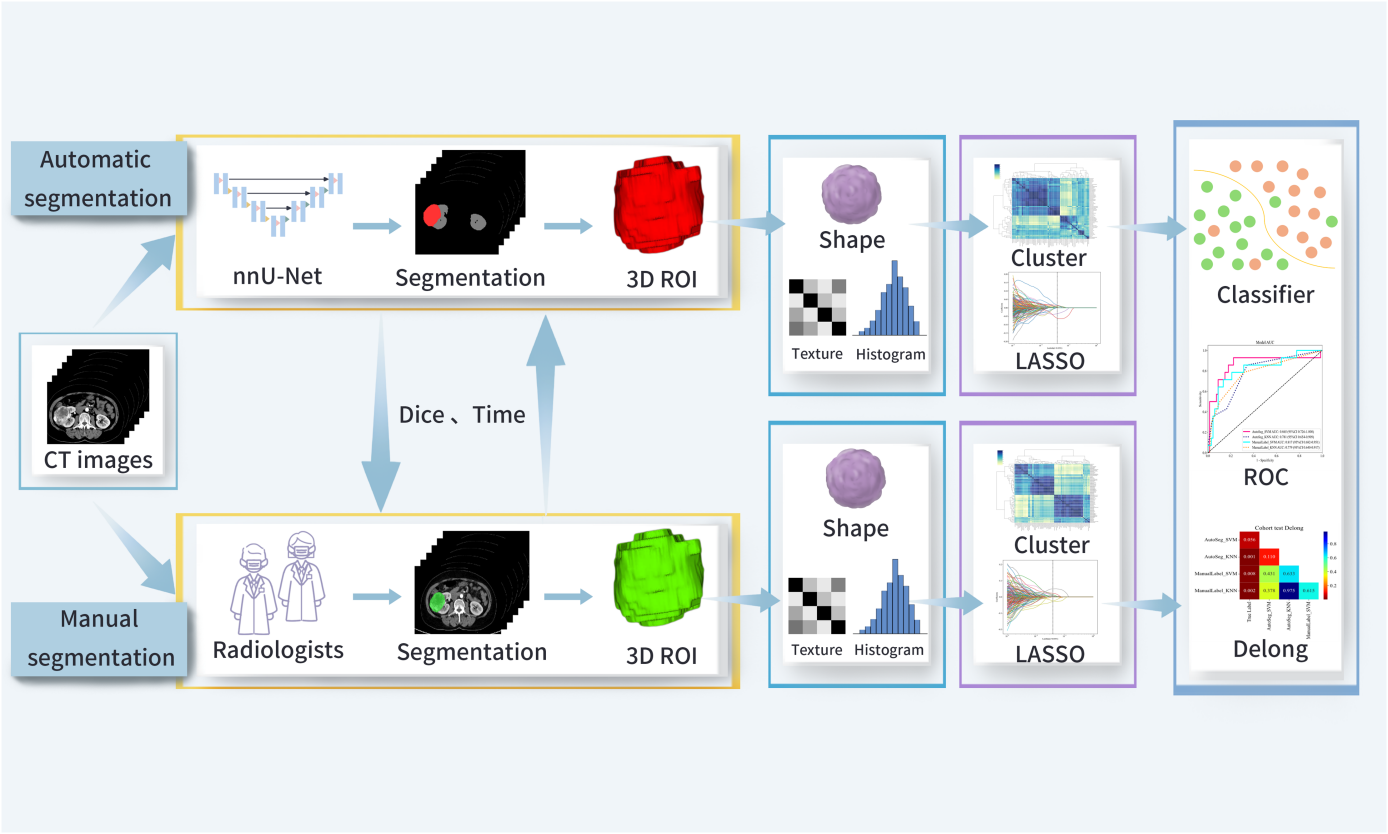
The study workflow. ROI, region of interest; Dice, Dice similarity coefficient; LASSO, least absolute shrinkage and selection operator; ROC, receiver operating characteristic.

This retrospective study included patients with clear ccRCC. A total of 405 patients treated at a tertiary hospital between August 2012 and August 2023 were included. The inclusion criteria were: (1) consecutive adult patients; (2) underwent partial or radical nephrectomy; (3) pathologically confirmed ccRCC; and (4) complete abdominal contrast-enhanced computed tomography (CT) images obtained within 2 weeks prior to surgery. The exclusion criteria were: (1) patients who received chemotherapy or radiotherapy before surgery; and (2) poor image quality (low resolution, image distortion, and blur). All patients were randomly divided into a training (*n* = 324) and test (*n* = 81) cohorts in a 4:1 ratio. The sample size was determined based on the principle of “feature-to-sample matching,” in combination with references from similar studies. The study flowchart is shown in Figs. 1 and 2. The study was approved by the Institutional Review Board of the Second Affiliated Hospital of Anhui Medical University [No. YX2024-219]. Given that this study is a retrospective analysis, informed consent was waived.

### CT acquisition

The study utilized preoperative contrast-enhanced CT scans, which included non-contrast, corticomedullary, parenchymal, and excretory phases. For subsequent tumor segmentation and radiomics analysis, the corticomedullary phase images were selected. This phase offers the clearest tumor boundaries due to the marked enhancement of ccRCC against the renal medulla and surrounding tissues, thereby facilitating accurate region-of-interest delineation. Moreover, the corticomedullary phase is widely adopted in recent radiomics studies for preoperative ccRCC grading, supporting its reliability and applicability in this analysis (Demirjian et al., 2022; Zheng et al., 2021).

### Tumor segmentation

**Figure 3:**
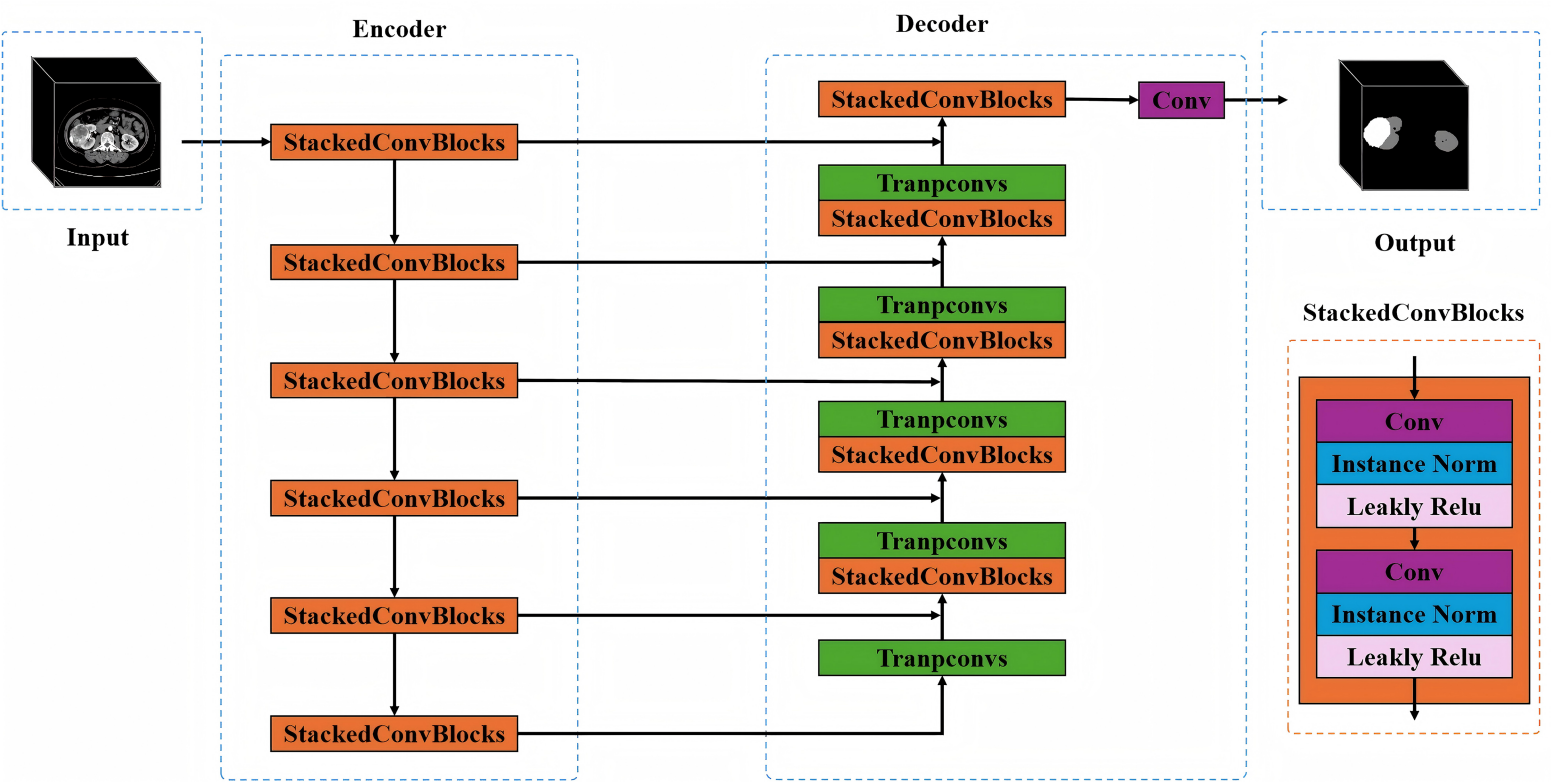
Schematic diagram of the core network architecture within the nnU-Net framework (based on the 3D U-Net design). The encoder uses stacked convolutional blocks with max pooling for downsampling. Each block has two layers with 3D convolutions, instance normalization, and leaky ReLU. The decoder mirrors the encoder, using transposed convolutions for upsampling. It concatenates and fuses features from the encoder, then outputs a segmentation map *via* a sigmoid convolutional layer. After fivefold cross-validation, the network segments kidneys and tumors, with the tumor mask as the final result.

Automatic segmentation: A pretrained nnU-Net model was used to segment corticomedullary phase CT images (Isensee et al., 2021). The nnU-Net model was pretrained on a publicly available dataset from the 2019 Kidney Tumor Segmentation Challenge (KiTS19, https://github.com/neheller/kits19) to segment kidneys and tumors (Heller et al., 2021; Sathianathen et al., 2021). This model was applied directly to our data without further fine-tuning. The configuration of the pretrained nnU-Net model, as adapted for the KiTS19 task, is as follows: nnU-Net is an adaptive deep learning framework for medical image segmentation proposed by the German Cancer Research Center. Its core philosophy is not to design a completely new network architecture, but rather to enable the traditional U-Net structure to automatically adjust its network configuration, preprocessing pipeline, training strategy, and postprocessing methods based on the specific task and dataset characteristics—thereby achieving optimal segmentation performance. Preprocessing: For the original images, the CT Hounsfield Unit values were initially clipped to a range of [−31, 257], and the z-score was normalized (standard score normalization) to a range of [0,1]. Data augmentation techniques were applied, including random cropping, rotation, scaling, flipping, Gaussian noise addition, and elastic deformation, to generate augmented inputs sized at 80 × 256 × 256 voxels (voxel spacing of 5 mm × 0.8 mm × 0.8 mm). Training settings for tumor segmentation: The Stochastic Gradient Descent optimizer was applied with an initial learning rate of 0.01, 1,000 total training iterations, and a batch size of 2. The target of segmentation in this study was the kidney tumor in CT images; therefore, the 3D U-Net network within the nnU-Net was chosen for model training. The structure of the 3D U-Net comprises four parts: Input, Encoder, Decoder, and Output (Fig. 3). The encoder comprises a series of stacked convolutional blocks with downsampling operations, where each block follows a standard two-layer convolutional structure. Each layer includes 3D convolutional units, instance normalization, and leaky ReLU activation functions, followed by max pooling for downsampling. The decoder consists of a series of stacked convolutional layers corresponding to the encoder and transposed convolutional upsampling operations. It first concatenates multiscale features from the encoder output with upsampled features, then uses stacked convolutional layers to further fuse these features, and finally applies a convolutional layer with a sigmoid function to output the segmentation probability map. After fivefold cross-validation, the network outputs the kidney and tumor, with the tumor segmentation mask extracted as the result.

Manual segmentation: The same pre-processing steps were applied to the CT images. Using ITK-SNAP v 3.8.0 (www.itksnap.org), two experienced radiologists (eight and 10 years of experience) manually outlined the tumor boundaries layer by layer. The delineation included the solid components of the tumor, cystic necrosis, capsule, intratumoral vessels, and calcified areas, excluding the perirenal and renal sinus fat, with a final three-dimensional region of interest. Before delineation, 30 cases were randomly chosen for independent segmentation by two radiologists. Two weeks later, one radiologist re-segmented the 30 images. Intra-and interclass correlation coefficients (ICC) were used to evaluate the stability, reproducibility, and robustness of the extracted features. Features with an ICC > 0.75 were considered to have agreeable reproducibility.

Comparison of time between automatic segmentation and manual delineation: (1) Thirty cases were randomly selected from the test cohort, and two radiologists, one with over 10 years of experience in diagnostic imaging and the other with <10 years, independently delineated the tumors on the cases displayed on a computer screen, with the time spent delineating each tumor recorded. (2) The same 30 tumors were segmented using nnU-Net, and the time taken was recorded.

### Feature extraction and selection

Feature extraction: A total of 1,834 features were extracted from the automatic and manual label segmentation results using the Pyradiomics package. The features include: (1) first-order features; (2) shape features; and (3) texture features, which encompass the gray-level co-occurrence matrix, gray-level dependence matrix, gray-level run length matrix, gray-level size zone matrix, and neighborhood gray-tone difference matrix. A detailed description of all image-based features and the working principles of the pyradiomics package can be found online (https://pyradiomics.readthedocs.io/en).

Feature selection: (1) All extracted radiomic features were statistically analyzed using the Mann–Whitney U test, and only those with *P* < 0.05 were retained. (2) The Spearman rank correlation coefficient was used to eliminate redundant features; when the absolute value of the correlation coefficient between two features exceeded 0.9, one was retained. (3) A greedy recursive feature elimination strategy was employed to remove features with the highest redundancy from the current set at each step. (4) Feature dimensionality reduction was performed using the Least Absolute Shrinkage and Selection Operator (LASSO). The optimal regularization parameter (λ) was determined by 10-fold cross-validation to minimize the cross-validation error. Features with non-zero coefficients at this λ value were retained to form the selected feature subset. All feature selection steps were strictly restricted to the training cohort, and the selected features were subsequently applied to the test cohort without modification to prevent data leakage.

### Prediction model construction and evaluation

Prediction models for WHO/ISUP grading were constructed using the Support Vector Machine (SVM) and K-nearest neighbors (KNN) algorithms, including the AutoSeg-SVM, AutoSeg-KNN, ManualLabel-SVM, and ManualLabel-KNN models. The models were designed to predict the binary outcome of low-grade (WHO/ISUP grades I–II) *vs*. high-grade (WHO/ISUP grades III–IV) ccRCC. Five-fold cross-validation was used for parameter tuning with multiple training iterations to determine the optimal hyperparameters for each classifier. The performance of each model was compared using the receiver operating characteristic (ROC) curve and the area under the curve (AUC). Furthermore, the accuracy, sensitivity, and specificity were calculated to evaluate the performance of each model in the classification task.

### Statistical analysis

Statistical analyses were performed using SPSS 25.0. Continuous data were presented as mean ± standard deviation, while categorical data were expressed as counts and percentages (%). To compare the means between groups, either the Student’s independent t-test or the Mann–Whitney U test was employed, depending on normality assumptions. Categorical data were analyzed using the chi-square test or Fisher exact test, as appropriate, with the latter being used when the expected frequency was ≤5. Statistical significance was set at *P* < 0.05. The Dice similarity coefficient (DSC) was used to assess the performance of the automatic segmentation model in terms of segmentation.

## Results

### Baseline patient characteristics

The 405 enrolled patients were randomly assigned to either a training cohort (*n* = 324) or test cohort (*n* = 81) in a 4:1 ratio. Table 1 summarizes the baseline clinical characteristics of both groups.

**Table 1:** Baseline characteristics of the patient.

Characteristic	Training cohort (*n* = 324)	Test Cohort (*n* = 81)	*p*
Age (years)	57.19 ± 13.02	59.80 ± 11.31	0.10
Gender			0.53
Male	208 (64.2%)	55 (67.9%)	
Female	116 (35.8%)	26 (32.1%)	
WHO/ISUP grade			0.30
Grade I	76 (23.5%)	28 (34.6%)	
Grade II	195 (60.2%)	40 (49.4%)	
Grade III	39 (12.0%)	8 (9.9%)	
Grade IV	14 (4.3%)	5 (6.2%)	
T stage			0.95
T1	226 (69.8%)	56 (69.1%)	
T2	48 (14.8%)	13 (16.0%)	
T3	49 (15.1%)	12 (14.8%)	
T4	1 (0.3%)	0 (0%)	
N stage			1.00
N0	308 (95.1%)	77 (95.1%)	
N1	16 (4.9%)	4 (4.9%)	
M stage			0.22
M0	318 (98.1%)	80 (98.8%)	
M1	6 (1.9%)	1 (1.2%)	
Maximum tumor diameter (mm)	51.90 ± 23.61	54.27 ± 30.82	0.52

**Note:**

WHO/ISUP, World Health Organization/International Society of Urological Pathology.

### Performance of tumor segmentation

The comparison of nnU-Net model tumor segmentation with manual annotations showed a DSC of 0.842 ± 0.149. Figure S1 shows examples comparing nnU-Net with radiologist’s manual segmentation. In this study, ccRCC tumors were precisely segmented using nnU-Net, demonstrating that the automatic segmentation model exhibits good segmentation performance and strong robustness, and can provide high-quality image input for subsequent grading prediction models. A comparison of the times required for tumor segmentation revealed that the average time for nnU-Net was (1.29 ± 0.16) min per tumor, while the average time for radiologists was (7.07 ± 2.01) min. This difference was statistically significant (t = −15.54, *P* < 0.01).

### Feature extraction and selection

**Figure 4:**
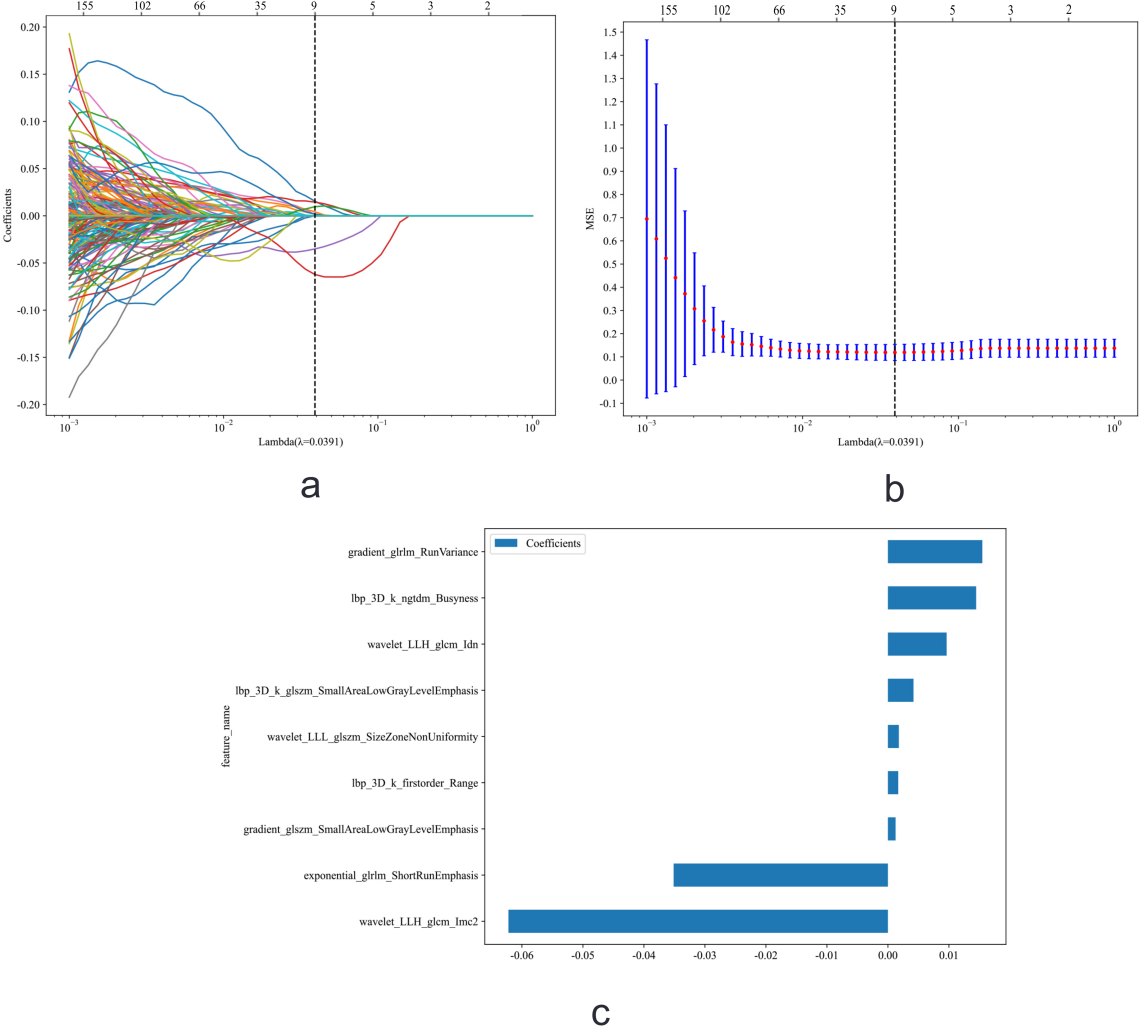
Feature selection using Least Absolute Shrinkage and Selection Operator (LASSO) regression. (A) Coefficient paths of all radiomic features across a sequence of log(λ) values. The upper x-axis indicates the average number of non-zero coefficients at each λ. (B) Ten-fold cross-validation mean squared error (MSE) curve for tuning the regularization parameter λ. The dashed vertical line marks the optimal λ value selected (lambda.1se, the largest λ within one standard error of the minimum MSE), which was used for final model selection to promote sparsity and generalizability. (C) Coefficients of the nine non-zero features retained.

A total of 1,834 features were extracted from each tumor in the training cohort of 324 patients. After LASSO Regression, nine features were retained as subsets for automatic segmentation (Fig. 4). In the manual segmentation group, nine features were also selected (Fig. S2).

### Performance and evaluation of the model

**Figure 5:**
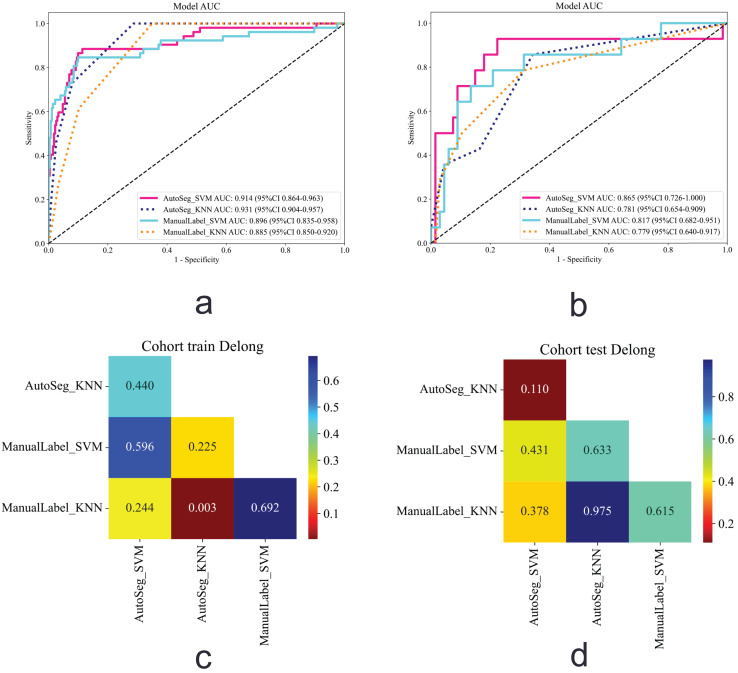
ccRCC classification results. (A) ROC curve in the training cohort. (B) ROC curve in the test cohort. (C) Heatmap showing the pairwise comparison of AUCs among different models using a two-tailed DeLong test in the training cohort. (D) Heatmap showing the pairwise comparison of AUCs among different models using a two-tailed DeLong test in the test cohort. ccRCC, Clear Cell Renal Cell Carcinoma; ROC, Receiver Operating Characteristic.

The SVM and KNN algorithms were used to build the AutoSeg-SVM, AutoSeg-KNN, ManualLabel-SVM, and ManualLabel-KNN models based on the extracted features. The ROC curves for the training and testing cohorts are shown in Fig. 5. The performance metrics of the model are shown in Table 2. The AutoSeg-SVM model achieved the highest AUC value of 0.865 (0.726–1.000), with 79% accuracy, 85.7% sensitivity, and 77.6% specificity, outperforming the other three models. The ManualLabel-SVM model achieved an AUC of 0.817 (0.682–0.951) in the test cohort, with 82.7% accuracy, 64.3% sensitivity, and 86.6% specificity, outperforming the ManualLabel-KNN model. AutoSeg-SVM and AutoSeg-KNN outperformed their manually labeled counterparts (AutoSeg-SVM > ManualLabel-SVM, AutoSeg-KNN > ManualLabel-KNN). To compare the performance of the automatic and manual segmentation–based models, the DeLong test was employed. The results indicated that there was no significant difference between the AutoSeg-SVM model and ManualLabel-SVM model in the test cohort (*P* = 0.431). Among all models, the AutoSeg-SVM model demonstrated the highest AUC and thus the best overall performance.

**Table 2:** Performances of prediction models using different segmentation methods.

Performance		AutoSeg		ManualLabel	
		SVM	KNN	SVM	KNN
Training cohort	AUC	0.914	0.931	0.896	0.885
	95% CI	[0.864–0.963]	[0.904–0.957]	[0.835–0.958]	[0.850–0.920]
	Accuracy	0.883	0.889	0.889	0.852
	Sensitivity	0.865	0.731	0.827	0.615
	Specificity	0.886	0.919	0.901	0.897
Test cohort	AUC	0.865	0.781	0.817	0.779
	95% CI	[0.726–1.000]	[0.654–0.909]	[0.682–0.951]	[0.640–0.917]
	Accuracy	0.790	0.765	0.827	0.827
	Sensitivity	0.857	0.429	0.643	0.500
	Specificity	0.776	0.836	0.866	0.896

**Note:**

AUC, Area Under the Curve; CI, Confidence Interval.

**Figure 6:**
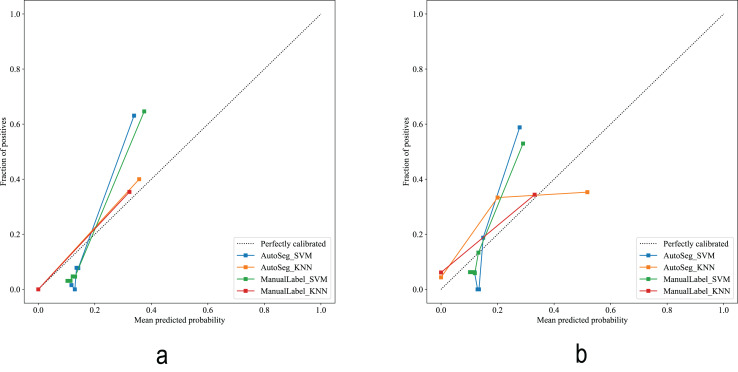
Calibration curves of the predictive models. (A) Calibration curve in the training cohort. (B) Calibration curve in the test cohort.

**Figure 7:**
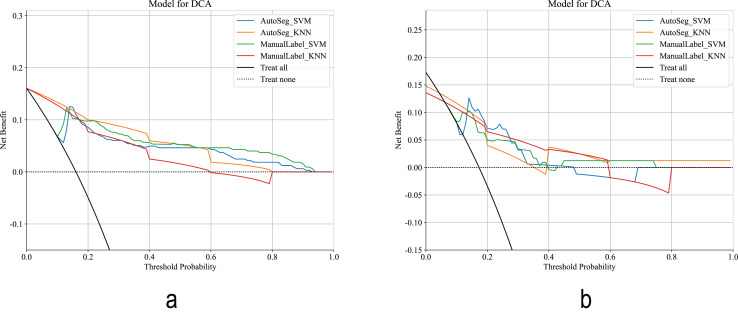
Decision curve. (A) Decision curve in the training cohort. (B) Decision curve in the test cohort.

The calibration curves (Fig. 6) showed that the AutoSeg-SVM and ManualLabel-SVM models were nearly overlapping, indicating that automatic segmentation did not compromise the accuracy of probability predictions. Decision curve (Fig. 7) analysis further confirmed that AutoSeg-SVM provided comparable clinical net benefit to ManualLabel-SVM. These findings, from both the perspectives of probabilistic accuracy and clinical decision value, strongly support that automatic segmentation can maintain model performance while achieving equivalent clinical applicability to manual segmentation, suggesting its potential as an effective substitute.

## Discussion

In this study, the pretrained nnU-Net model was applied for ccRCC tumor segmentation (DSC = 84.2%), reflecting its impressive segmentation accuracy. We compared an automatic segmentation model based on deep learning to a prediction model constructed using manual segmentation methods. The results indicated that the AutoSeg-SVM model exhibited the best performance. The prediction model’s performance in the automatic segmentation group was comparable to, and in some cases exceeded, the manually labeled results.

This study conducted a comparative analysis of time efficiency between automatic segmentation and manual delineation, revealing that automatic segmentation saved an average of 5.78 min per case (*P* < 0.01). This finding not only highlights the efficiency advantage of automatic segmentation but also suggests its potential applicability in high-throughput radiomics research and clinical batch processing. Particularly in multicenter studies or large-scale cohorts, automatic segmentation can significantly reduce labor costs and enhance workflow efficiency. We selected 30 cases for time comparison, aiming to cover a range of tumor sizes and morphologies to ensure representativeness in time evaluation. Preliminary analysis showed a significant time difference between the two methods (*P* < 0.01), with relatively stable standard deviations in time measurements, indicating that a sample size of 30 was sufficient to detect a meaningful efficiency gap. Future studies will expand the sample size and incorporate segmentation quality and model performance for a more comprehensive evaluation.

Although the extracted features from the two segmentation methods were entirely different, from a clinical application perspective, automatic segmentation remains a valid and valuable alternative—as long as models based on it demonstrate comparable or superior performance in preoperative grading prediction compared to those based on manual segmentation. The divergence in feature sets may stem from the inherent differences in segmentation logic: manual segmentation relies on the radiologist’s subjective visual judgment, whereas deep learning models such as nnU-Net depend on objective computations of CT values (HU) and texture continuity. This allows the model to capture subtle features related to tumor heterogeneity that may be imperceptible to the human eye. This phenomenon is analogous to how different pathological staining techniques (*e.g*., H&E staining *vs*. immunohistochemistry) reveal distinct biological aspects of the same tumor, yet both contribute to diagnosis. Therefore, the difference in feature sets does not undermine the value of automatic segmentation as a substitute. The key lies in its ability to achieve diagnostic conclusions equivalent to manual segmentation while maintaining high geometric accuracy and significantly improving efficiency—laying a solid foundation for its application in clinical practice and large-scale research.

From the perspective of clinical needs, the advantages of automatic segmentation are even more pronounced. In terms of technical performance, its accuracy and efficiency are not influenced by operator experience, offering greater stability compared to manual segmentation. Clinically, automatic segmentation reduces the time required per case by 5.78 min compared to manual segmentation (*P* < 0.01); assuming 200 cases per year, this translates to approximately 19.3 h of saved workload. Moreover, it eliminates reliance on highly experienced specialists, making it easier to implement in primary care settings and well-suited for large-scale studies or urgent preoperative grading scenarios. In terms of research scalability, automatic segmentation can capture subtle features—such as gray-level run-length matrix patterns—that are often imperceptible to the human eye, potentially offering new insights into the molecular mechanisms of ccRCC. In summary, despite differences in feature sets and modeling approaches, automatic segmentation aligns well with clinical demands and holds strong potential to replace manual segmentation for preoperative grading of ccRCC.

This study chose to evaluate the value of automatic segmentation by constructing grading models based on the segmented regions, rather than merely comparing tumor volume or segmentation time. This decision was driven by the clinical need for preoperative grading of clear cell renal cell carcinoma (ccRCC): whether automatic segmentation can improve efficiency without compromising predictive performance. Specifically, comparing only volume or time has limitations. Tumor volume reflects size but not the internal characteristics critical for grading, such as enhancement heterogeneity of solid components or necrotic regions—features that cannot be captured by volume alone. Even if volumes match, automatic segmentation may miss these subtle features, leading to grading bias that volume comparison alone cannot detect. Modeling is key to validating performance. By constructing identical SVM models using regions derived from both segmentation methods, we compared their grading performance (*e.g*., AutoSeg-SVM AUC = 0.865 *vs*. ManualLabel-SVM AUC = 0.817; DeLong test *P* > 0.05), directly demonstrating that automatic segmentation maintains predictive reliability while saving time. This core conclusion cannot be drawn from volume or ROI comparisons alone.

Tumor segmentation is a fundamental aspect of renal tumor radiomics research (Yang et al., 2023). Manual segmentation is often limited by operator experience or subjective judgment. Automatic segmentation can extract precise tumor boundaries and morphological information, comprehensively capturing the spatial heterogeneity of tumors, and uncovering features that are difficult for the human eye to detect, resulting in accurate segmentation (Klontzas et al., 2024). The proposed model can automatically adjust the network structure and training parameters based on the geometric structure of the input images and dataset characteristics, achieving optimal segmentation results. In the 2019 KiTS Challenge, nnU-Net won the title with an average Dice value of 0.974 (kidney) and 0.851 (tumor), verifying its advantage in the segmentation of complex anatomical structures. Its automatic design has greatly reduced the threshold of algorithm development and become a benchmark tool for medical image segmentation. The nnU-Net reduced manual intervention, optimized the omics workflow, simplified the complexity of medical image segmentation, and achieved automation, thereby reducing labor costs. Automatic segmentation may provide more consistent tumor boundaries and capture subtle morphological features that are challenging to delineate manually, potentially contributing to its comparable or slightly better performance.

Conventionally, it has been held that accurate ROI segmentation is the basis for the reliability of radiomics features. Traditional manual segmentation, performed by radiologists to identify tumor boundaries, is accurate but time-consuming, which limits its use in large-scale cohort studies. In recent years, numerous studies have employed open-source deep learning frameworks for automated segmentation to extract radiomic features. However, current automated segmentation techniques cannot achieve perfect spatial congruence with lesion regions manually delineated by radiologists. Despite this critical limitation, there has been a paucity of empirical evidence demonstrating whether the discrepancies between automated segmentation and radiologist-defined manual delineation significantly impact the extraction of radiomic features and, consequently, the predictive performance of constructed models. This knowledge gap raises important questions about the reliability and clinical applicability of radiomics-based approaches that rely solely on automated segmentation methods.

The nnU-Net model demonstrated exceptional performance and unique advantages in the field of medical image segmentation due to its excellent adaptability. The impact of tumor segmentation accuracy on tumor grading in the context of clinical concerns regarding prognosis and cancer management has been poorly investigated. The pathological nuclear grade of ccRCC is closely associated with prognosis. Accurate preoperative prediction of the WHO/ISUP grade can help guide treatment and monitoring strategies for patients, avoiding unnecessary surgical risks (Li et al., 2024; Nie et al., 2023). Radiomics, a noninvasive technique, extracts a vast array of features from imaging to analyze tumor heterogeneity and provides crucial information for tumor prognosis (Demirjian et al., 2022; Yin et al., 2022). The performance of the prediction model in the automatic segmentation group was comparable to that of the manual labeling results, even exceeding it in some cases. This indicates the feasibility of using automatic segmentation methods for predicting pathological grading, with the potential to replace traditional manual segmentation methods in clinical practice. Therefore, this study compared radiomic feature extraction and model construction using both segmentation approaches. The results demonstrated that models based on manual segmentation achieved AUC values of 0.817 and 0.779, while those from automated segmentation yielded AUC of 0.865 and 0.781, respectively. DeLong test revealed no statistically significant differences between the models (*P* > 0.05), indicating that the predictive performance of automated segmentation closely approximated that of radiologist-defined manual segmentation. To further interpret this result, we conducted a power analysis based on the observed AUC difference (0.048) and an estimated standard error of approximately 0.07. The analysis revealed that, given the current sample size (test set *n* = 81), the study had only about 11% power to detect this difference—substantially lower than the commonly recommended threshold of 80%. This indicates that the study lacks sufficient statistical power to detect small effect sizes, which may obscure potential performance differences.

Furthermore, we conducted a comprehensive time efficiency analysis comparing both segmentation approaches. The results demonstrated that automated segmentation significantly reduced processing time compared to manual delineation, with an average time saving of 5.78 min per tumor (*P* < 0.01). These findings substantiate that the automated segmentation method not only achieves comparable predictive performance to radiologist-defined manual segmentation but also offers substantial time efficiency advantages. This approach effectively reduces labor costs and enhances research productivity, thereby providing an efficient solution for high-throughput feature extraction in subsequent radiomics studies. This meets the requirements of the “standardized radiomics workflow” proposed by van Griethuysen et al. (2017).

This study has several limitations that warrant consideration: (1) The current analysis was restricted to arterial phase images, potentially underutilizing available data. Future investigations will extend to venous and excretory phase CT images of ccRCC to validate whether similar conclusions can be drawn across different contrast phases. (2) The segmentation was confined to tumor regions without including the entire kidney or perirenal fat, which may limit comprehensive characterization of tumor microenvironment. Subsequent studies should incorporate whole-kidney and perirenal segmentation to enable more thorough tumor analysis. (3) The single-center, retrospective nature of this dataset may constrain the model’s generalizability. To address this limitation, we plan to conduct multicenter studies with expanded sample sizes to enhance the model’s generalizability and clinical applicability. (4) To further assess the model’s discrimination and clinical utility, we plan to conduct core subgroup analyses and a prospective clinical validation. Specifically, we will evaluate three key clinical subgroups—tumor size (small, regular, large), clinical risk (presence or absence of comorbidities), and tumor location (upper, mid, lower pole)—by calculating AUC, sensitivity, and specificity for each, and applying the DeLong test to compare performance across groups. This will help determine the model’s stability in diverse clinical scenarios. Additionally, we will initiate a prospective clinical observation involving 100 newly diagnosed ccRCC patients to validate the model’s predictive value in guiding real-world treatment decisions.

## Conclusions

The nnU-Net automatic segmentation showed comparable diagnostic efficacy to manual segmentation, with a trend toward better performance (AUC=0.865 *vs*. 0.817), and significantly reduced the time required for segmentation (1.29 min *vs*. 7.07 min). This model optimizes radiomics workflow, alleviates the burden on radiologists in daily measurement tasks, and enhances work efficiency. Accurately predicting ccRCC pathological grade before surgery helps to prevent unnecessary overtreatment, allowing for more precise individualized treatment plans, and facilitating a more accurate prognostic assessment.

## Supplemental Information

10.7717/peerj.21022/supp-1 Supplemental Information 1 STROBE checklist.

10.7717/peerj.21022/supp-2 Supplemental Information 2 Code for segmentation and modeling.

10.7717/peerj.21022/supp-3 Supplemental Information 3 Supplementary Figures.

10.7717/peerj.21022/supp-4 Supplemental Information 4 Raw data. Clinical data = Case clinical baseline data.DICE = Similarity of pre-trained nnunet model segmentation of in-house tumor CT images. Time comparison = Time spent on automatic *vs*. manual segmentation.

10.7717/peerj.21022/supp-5 Supplemental Information 5 Codebook for clinical data.

## List of abbreviations

ccRCC: Clear Cell Renal Cell Carcinoma
ICC: Intraclass Correlation Coefficient
ROC: Receiver Operating Characteristic
AUC: Area Under the Curve
WHO/ISUP: World Health Organization/International Society of Urological Pathology
DSC: Dice Similarity Coefficient
LASSO: Least Absolute Shrinkage and Selection Operator

## Additional Information and Declarations

### Data Availability

The following information was supplied regarding data availability:

The raw measurements and code are available in the Supplemental Files.

The code is also available at GitHub: https://github.com/neheller/kits19.
