# Supplementary material for: Application of adaptive deep learning-based automatic segmentation in radiomics model for preoperative WHO/ISUP grading of clear cell renal cell carcinoma: a retrospective comparative study with manual segmentation
Source: PeerJ. 2026 Mar 27;14:e21022. doi: 10.7717/peerj.21022 (PMC13034870; doi:10.7717/peerj.21022)
Supplement: Supplemental Information 1 [file peerj-14-21022-s001.docx]

STROBE Statement—checklist of items that should be included in reports of observational studies

|  | Item No. | Recommendation | Page  No. | Relevant text from manuscript |
| --- | --- | --- | --- | --- |
| **Title and abstract** | 1 | (*a*) Indicate the study’s design with a commonly used term in the title or the abstract | 1 | Application of adaptive deep learning-based automatic segmentation in radiomics model for preoperative WHO/ISUP grading of clear cell renal cell carcinoma: A comparative study with manual segmentation. |
|  |  | (*b*) Provide in the abstract an informative and balanced summary of what was done and what was found | 1,2 | This study analyzed CT images of 405 patients (training/test cohort, 324/81) with pathologically confirmed ccRCC. Two methods were used for tumor segmentation: (1) automatic segmentation: the nnU-Net model, trained on the public KiTS19 dataset, and (2) manual segmentation. Radiomics features were extracted and selected from both automatically and manually segmented images. Support vector machine (SVM) and K-nearest neighbors were used to construct pathological grade prediction models. The segmentation accuracies of nnU-Net and manual annotation were compared.  Conclusions: The nnU-Net automatic segmentation showed higher diagnostic efficacy than manual segmentation in preoperative WHO/ISUP grade prediction for ccRCC. It significantly reduced the time required for lesion segmentation and improved work flow efficiency. |
| Introduction | | | |  |
| Background/rationale | 2 | Explain the scientific background and rationale for the investigation being reported | 3 | Renal cancer has diverse histological classifications, with approximately 75% of cases being clear cell renal cell carcinoma (ccRCC).Studies have shown that pathological nuclear grading is an independent risk factor for tumor malignancy and postoperative recurrence.Radiomics, by transforming imaging data into a large amount of quantifiable data, delves into underlying lesion characteristics, offering potential advancement in tumor grade prediction research. |
| Objectives | 3 | State specific objectives, including any prespecified hypotheses | 4 | The aim of this study was to assess whether automatic segmentation versus manual delineation affects the WHO/ISUP grade prediction for ccRCC based on radiomics. |
| Methods | | | |  |
| Study design | 4 | Present key elements of study design early in the paper | 4 | In this study, a deep learning-based automatic segmentation model was used to segment ccRCC tumors and compare the results with the radiologist's manual delineation. |
| Setting | 5 | Describe the setting, locations, and relevant dates, including periods of recruitment, exposure, follow-up, and data collection | 4 | This retrospective study included patients with clear ccRCC. A total of 405 patients treated at a tertiary hospital between August 2012 and August 2023 were included. |
| Participants | 6 | (*a*) *Cohort study*—Give the eligibility criteria, and the sources and methods of selection of participants. Describe methods of follow-up  *Case-control study*—Give the eligibility criteria, and the sources and methods of case ascertainment and control selection. Give the rationale for the choice of cases and controls  *Cross-sectional study*—Give the eligibility criteria, and the sources and methods of selection of participants | 4 | The 2016 World Health Organization/International Society of Urological Pathology (WHO/ISUP) grade system classifieds ccRCC into four grades, with grade I and II categorized as low-grade tumors and grade III and IV as high-grade tumors.  The inclusion criteria were: (1) consecutive adult patients; (2) underwent partial or radical nephrectomy; (3) pathologically confirmed ccRCC; and (4) complete abdominal contrast-enhanced CT images obtained within two weeks prior to surgery. The exclusion criteria were: (1) non-ccRCC pathological histological type (2) lack of WHO/ISUP grade or grade obtained through renal biopsy; (3) patients who received chemotherapy or radiotherapy before surgery; and (4) poor image quality (low resolution, image distortion, and blur). The study flowchart is shown in Figures 1 and 2. |
|  |  | (*b*) *Cohort study*—For matched studies, give matching criteria and number of exposed and unexposed  *Case-control study*—For matched studies, give matching criteria and the number of controls per case |  |  |
| Variables | 7 | Clearly define all outcomes, exposures, predictors, potential confounders, and effect modifiers. Give diagnostic criteria, if applicable | 5-8 | The effectiveness of different methods for segmenting tumor.  Two methods were used for tumor segmentation  The baseline clinical characteristics  CT acquisition  Feature extraction and selection  Prediction Model Construction and Evaluation |
| Data sources/ measurement | 8* | For each variable of interest, give sources of data and details of methods of assessment (measurement). Describe comparability of assessment methods if there is more than one group | 4-9 | A total of 405 patients treated at a tertiary hospital between August 2012 and August 2023 were included.  The images were obtained from three CT scanners: Siemens SOMATOM Force CT, Siemens Definition AS40, and Philips Brilliance iCT.  Automatic segmentation: A pretrained nnU-Net automatic segmentation model was used to segment corticomedullary phase CT images.  Manual segmentation: The same pre-processing steps were applied to the CT images. Using ITK-SNAP v 3.8.0 (www.itksnap.org), two experienced radiologists (eight and 10 years of experience) manually outlined the tumor boundaries layer by layer.  Feature extraction: 1,834 features were extracted from the automatic and manual label segmentation results using the Pyradiomics package.  Support vector machine (SVM) and K-nearest neighbors were used to construct pathological grade prediction models. The segmentation accuracies of nnU-Net and manual annotation were compared. |
| Bias | 9 | Describe any efforts to address potential sources of bias | 4,7,8 | All patients were randomly divided into a training (n = 324) and test (n = 81) cohorts in a 4:1 ratio.  Before delineation, 30 cases were randomly chosen for independent segmentation by two radiologists. Two weeks later, one radiologist re-segmented the 30 images. Intra-and interclass correlation coefficients (ICC) were used to evaluate the stability.  Breathing training was conducted before the examination to reduce artifact interference. |
| Study size | 10 | Explain how the study size was arrived at | 4 | Collect imaging data from a tertiary hospital from 2012 to 2023 and screen according to the corresponding inclusion and exclusion criteria. |

Continued on next page

| Quantitative variables | 11 | Explain how quantitative variables were handled in the analyses. If applicable, describe which groupings were chosen and why | 8,9 | Continuous data were presented as mean ± standard deviation, while categorical data were expressed as counts and percentages (%). To compare the means between groups, either the Student's independent t-test or the Mann–Whitney U test was employed, depending on normality assumptions. Categorical data were analyzed using the chi-square test or Fisher exact test, as appropriate, with the latter being used when the expected frequency was ≤5. Statistical significance was set at p < 0.05.. The Dice similarity coefficient (DSC) was used to assess the performance of the automatic segmentation model in terms of segmentation. |
| --- | --- | --- | --- | --- |
| Statistical methods | 12 | (*a*) Describe all statistical methods, including those used to control for confounding | 7-9 | Intra-and interclass correlation coefficients (ICC) were used to evaluate the stability, reproducibility, and robustness of the extracted features.  Feature dimensionality reduction was performed using the Least Absolute Shrinkage and Selection Operator (LASSO).  Statistical analyses were performed using SPSS 25.0. To compare the means between groups, either the Student's independent t-test or the Mann–Whitney U test was employed, depending on normality assumptions. Categorical data were analyzed using the chi-square test or Fisher exact test, as appropriate, with the latter being used when the expected frequency was ≤5. Statistical significance was set at p < 0.05.. The Dice similarity coefficient (DSC) was used to assess the performance of the automatic segmentation model in terms of segmentation. |
|  |  | (*b*) Describe any methods used to examine subgroups and interactions | 8-9 | Categorical data were analyzed using the chi-square test or Fisher exact test, as appropriate, with the latter being used when the expected frequency was ≤5. Statistical significance was set at p < 0.05. |
|  |  | (*c*) Explain how missing data were addressed |  |  |
|  |  | (*d*) *Cohort study*—If applicable, explain how loss to follow-up was addressed  *Case-control study*—If applicable, explain how matching of cases and controls was addressed  *Cross-sectional study*—If applicable, describe analytical methods taking account of sampling strategy |  |  |
|  |  | (*e*) Describe any sensitivity analyses |  |  |
| Results | | | | |
| Participants | 13* | (a) Report numbers of individuals at each stage of study—eg numbers potentially eligible, examined for eligibility, confirmed eligible, included in the study, completing follow-up, and analysed | 4 | The 405 enrolled patients were randomly assigned to either a training cohort (n = 324) or test cohort (n = 81) in a 4:1 ratio. |
|  |  | (b) Give reasons for non-participation at each stage | 4 | The exclusion criteria were: (1) non-ccRCC pathological histological type (2) lack of WHO/ISUP grade or grade obtained through renal biopsy; (3) patients who received chemotherapy or radiotherapy before surgery; and (4) poor image quality (low resolution, image distortion, and blur). |
|  |  | (c) Consider use of a flow diagram | 4 | Figures 1 |
| Descriptive data | 14* | (a) Give characteristics of study participants (eg demographic, clinical, social) and information on exposures and potential confounders | 9 | Table 1 summarizes the baseline clinical characteristics of both groups. |
|  |  | (b) Indicate number of participants with missing data for each variable of interest |  |  |
|  |  | (c) *Cohort study*—Summarise follow-up time (eg, average and total amount) | 4 | between August 2012 and August 2023 |
| Outcome data | 15* | *Cohort study*—Report numbers of outcome events or summary measures over time | 9-10 | The comparison of nnU-Net model tumor segmentation with manual annotations showed a DSC of 0.842±0.149.  A comparison of the times required for tumor segmentation (Table 2) revealed that the average time for nnU-Net was (1.29±0.16) min per tumor, while the average time for radiologists was (7.07±2.01) min. This difference was statistically significant (t=-15.54, P<0.01). |
|  |  | *Case-control study—*Report numbers in each exposure category, or summary measures of exposure |  |  |
|  |  | *Cross-sectional study—*Report numbers of outcome events or summary measures |  |  |
| Main results | 16 | (*a*) Give unadjusted estimates and, if applicable, confounder-adjusted estimates and their precision (eg, 95% confidence interval). Make clear which confounders were adjusted for and why they were included |  |  |
|  |  | (*b*) Report category boundaries when continuous variables were categorized |  |  |
|  |  | (*c*) If relevant, consider translating estimates of relative risk into absolute risk for a meaningful time period |  |  |

| Other analyses | 17 | Report other analyses done—eg analyses of subgroups and interactions, and sensitivity analyses | 9,10, | After LASSO Regression, 9 features were retained as subsets for automatic segmentation(Figure 4). In the manual segmentation group, 9 features were also selected(Supplementary Figure 2).  The AutoSeg-SVM model achieved the highest AUC value of 0.865 (0.7259 - 1.0000), with 79% accuracy, 85.7% sensitivity, and 77.6% specificity, outperforming the other three models. |
| --- | --- | --- | --- | --- |
| Discussion | | | | |
| Key results | 18 | Summarise key results with reference to study objectives | 11 | In this study, the pretrained nnU-Net model was applied for ccRCC tumor segmentation (DSC=84.2%), reflecting its impressive segmentation accuracy. We compared an automatic segmentation model based on deep learning to a prediction model constructed using manual segmentation methods. The results indicated that the AutoSeg-SVM model exhibited the best performance. The prediction model's performance in the automatic segmentation group was comparable to, and in some cases exceeded, the manually labeled results. |
| Limitations | 19 | Discuss limitations of the study, taking into account sources of potential bias or imprecision. Discuss both direction and magnitude of any potential bias | 13,14 | This study has several limitations that warrant consideration: (1) The current analysis was restricted to arterial phase images, potentially underutilizing available data. (2) The segmentation was confined to tumor regions without including the entire kidney or perirenal fat, which may limit comprehensive characterization of tumor microenvironment. (3) The single-center, retrospective nature of this dataset may constrain the model's generalizability. |
| Interpretation | 20 | Give a cautious overall interpretation of results considering objectives, limitations, multiplicity of analyses, results from similar studies, and other relevant evidence | 14 | The automatic segmentation model based on the adaptive deep learning framework nnU-Net demonstrated better performance in predicting ccRCC pathological grading than manual label models. This model optimizes radiomics workflow, alleviates the burden on radiologists in daily measurement tasks, and enhances work efficiency. Accurately predicting ccRCC pathological grade before surgery helps to prevent unnecessary overtreatment, allowing for more precise individualized treatment plans, and facilitatig a more accurate prognostic assessment. |
| Generalisability | 21 | Discuss the generalisability (external validity) of the study results |  |  |
| Other information | |  | | |
| Funding | 22 | Give the source of funding and the role of the funders for the present study and, if applicable, for the original study on which the present article is based | Title page | This work was supported by University Natural Science Research Project of Anhui Province (2024AH050796) and the Research Fund of the Anhui Institute of Translational Medicine (2021zhyx-C45). |

*Give information separately for cases and controls in case-control studies and, if applicable, for exposed and unexposed groups in cohort and cross-sectional studies.

**Note:** An Explanation and Elaboration article discusses each checklist item and gives methodological background and published examples of transparent reporting. The STROBE checklist is best used in conjunction with this article (freely available on the Web sites of PLoS Medicine at http://www.plosmedicine.org/, Annals of Internal Medicine at http://www.annals.org/, and Epidemiology at http://www.epidem.com/). Information on the STROBE Initiative is available at www.strobe-statement.org.
