# Supplementary figures and images for: Application of adaptive deep learning-based automatic segmentation in radiomics model for preoperative WHO/ISUP grading of clear cell renal cell carcinoma: a retrospective comparative study with manual segmentation
Source: PeerJ. 2026 Mar 27;14:e21022. doi: 10.7717/peerj.21022 (PMC13034870; doi:10.7717/peerj.21022)

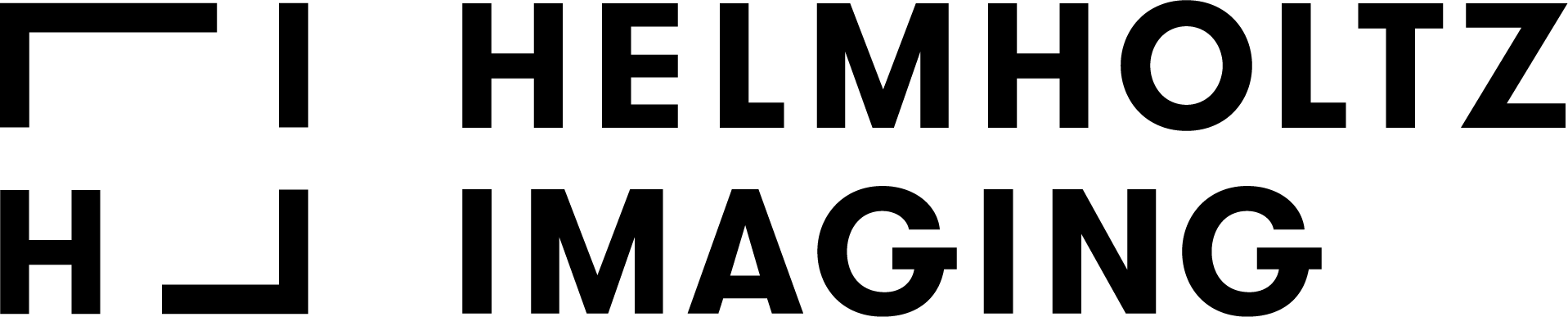

Supplement: Supplemental Information 2 [file peerj-14-21022-s002.zip › nnUNet/HI_Logo.png]
