# Supplementary material for: Application of adaptive deep learning-based automatic segmentation in radiomics model for preoperative WHO/ISUP grading of clear cell renal cell carcinoma: a retrospective comparative study with manual segmentation
Source: PeerJ. 2026 Mar 27;14:e21022. doi: 10.7717/peerj.21022 (PMC13034870; doi:10.7717/peerj.21022)
Supplement: Supplemental Information 3 [file peerj-14-21022-s003.docx]

**Supplementary Figure 1: Comparative Examples of nnU-Net (red) and Radiologist Manual Segmentation (green)**

**
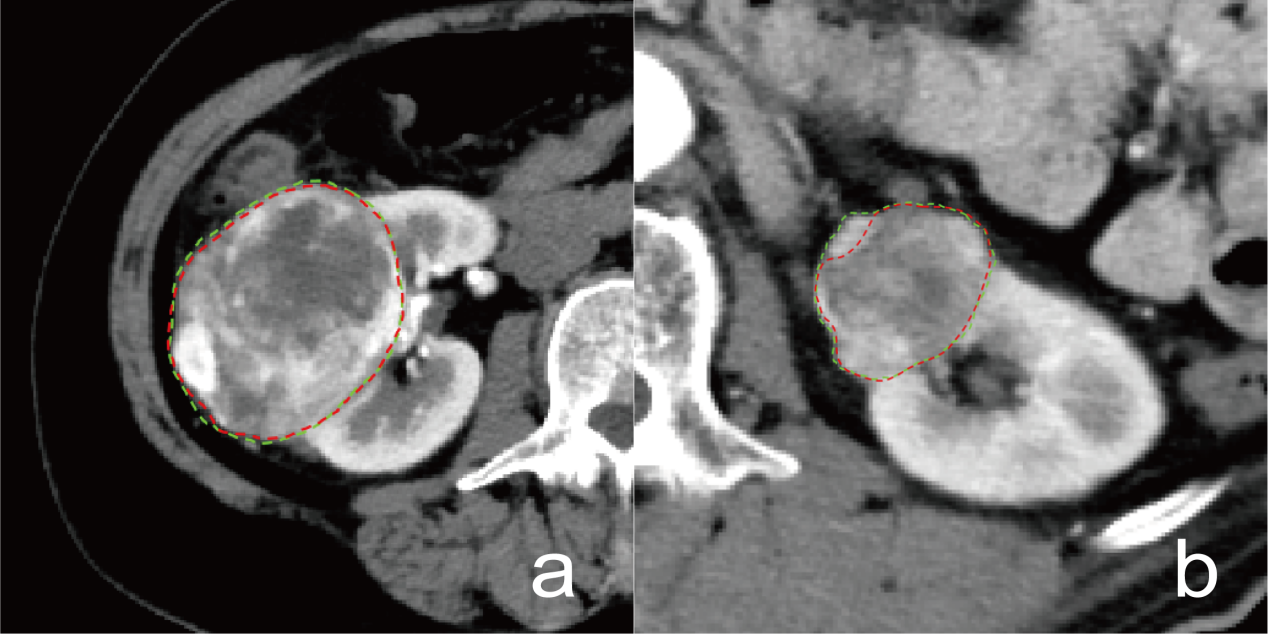
**

1. Case 1 (DSC=0.935): High concordance between nnU-Net prediction (red) and radiologist annotation (green). (b) Case 2 (DSC=0.844): Underestimation of solid tumor components in nnU-Net segmentation (red) compared to radiologist manual delineation (green). DSC: Dice Similarity Coefficient

**Supplementary Figure 2: LASSO Regression Analysis in Manual Segmentation of Medical Image**.


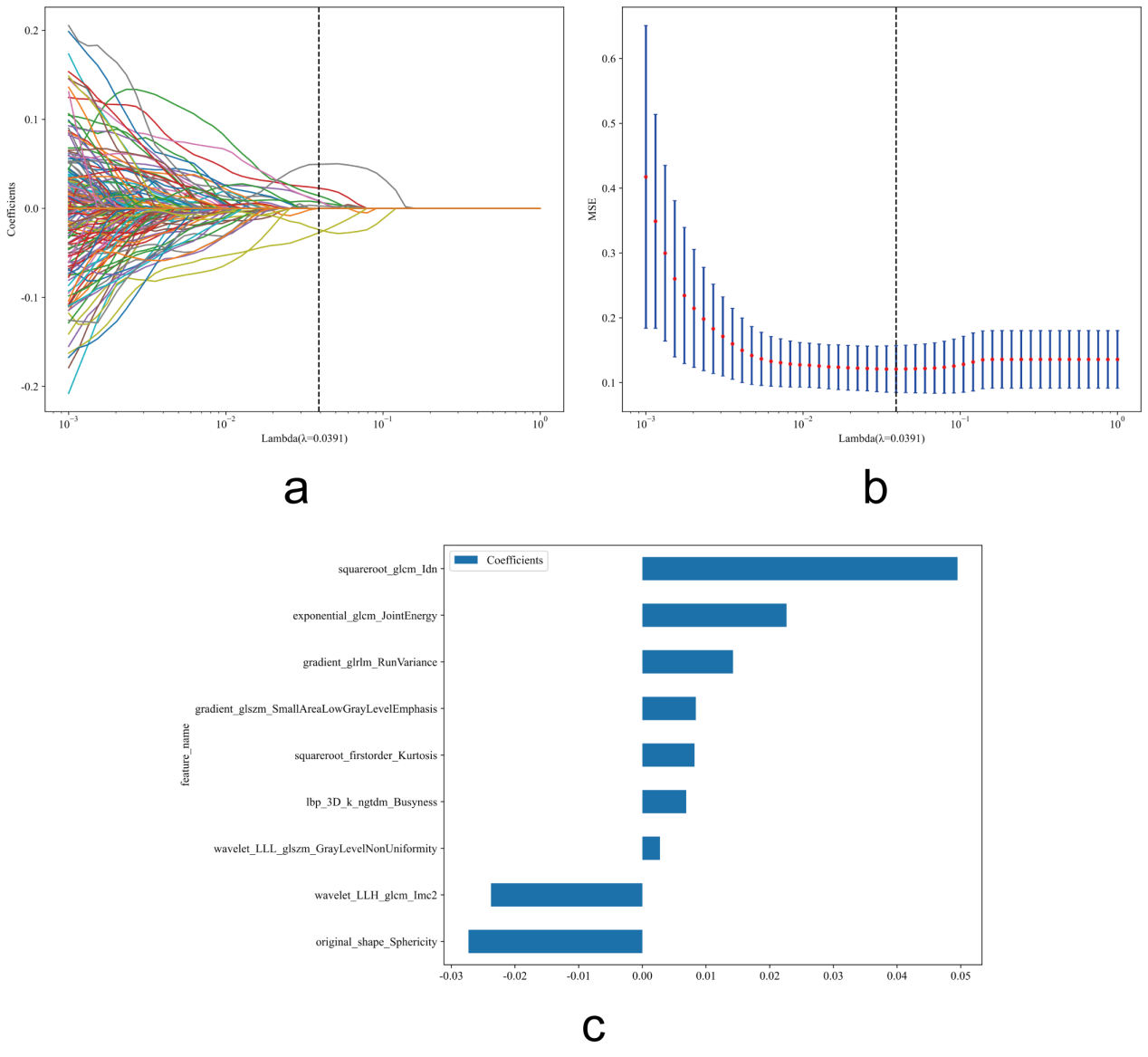


(a) LASSO regression. (b) MSE weight map. (c) feature coefficients. LASSO: Least Absolute Shrinkage and Selection Operator
