# Supplementary material for: Application of adaptive deep learning-based automatic segmentation in radiomics model for preoperative WHO/ISUP grading of clear cell renal cell carcinoma: a retrospective comparative study with manual segmentation
Source: PeerJ. 2026 Mar 27;14:e21022. doi: 10.7717/peerj.21022 (PMC13034870; doi:10.7717/peerj.21022)
Supplement: Supplemental Information 5 [file peerj-14-21022-s005.docx]

**Codebook for clinical data**

**Label**

0 = low grade

1 = high grade

**Gender**

0 = female

1 = male

**WHO/ISUP grade**

1 = Grade Ⅰ

2 = Grade Ⅱ

3 = Grade Ⅲ

4 = Grade Ⅳ
